# The Reticulocyte Hemoglobin Equivalent as a Screening Marker for Iron Deficiency and Iron Deficiency Anemia in Children

**DOI:** 10.3390/jcm10163506

**Published:** 2021-08-09

**Authors:** Vanessa Neef, Elke Schmitt, Peter Bader, Frank Zierfuß, Gudrun Hintereder, Andrea U. Steinbicker, Kai Zacharowski, Florian Piekarski

**Affiliations:** 1 Department of Anesthesiology, Intensive Care Medicine and Pain Therapy, University Hospital Frankfurt, Goethe University, 60590 Frankfurt, Germany; Vanessa.Neef@kgu.de (V.N.); elke.schmitt@kgu.de (E.S.); kai.zacharowski@kgu.de (K.Z.); 2 Department for Children and Adolescents, Division for Stem Cell Transplantation and Immunology, University Hospital Frankfurt, Goethe University, 60590 Frankfurt, Germany; peter.bader@kgu.de; 3 Central Laboratory, Centre of Internal Medicine, University Hospital Frankfurt, Goethe University, 60590 Frankfurt, Germany; Frank.Zierfuss@kgu.de (F.Z.); Gudrun.Hintereder@kgu.de (G.H.); 4 Department of Anesthesiology, Intensive Care and Pain Medicine, University Hospital Muenster, University of Muenster, 48149 Muenster, Germany; andrea.steinbicker@ukmuenster.de

**Keywords:** reticulocyte hemoglobin equivalent, iron deficiency, anemia, children

## Abstract

Background: Iron deficiency (ID) is one of the most common nutritional deficiencies in children worldwide and may result in iron deficiency anemia (IDA). The reticulocyte hemoglobin equivalent (Ret-He) provides information about the current availability of iron in erythropoiesis. This study aims to examine the validation of Ret-He as a screening marker for ID and IDA in children. Methods: Blood samples were retrospectively obtained from medical records. Anemia was defined according to the definition provided by the World Health Organization (WHO) for children. ID was defined by transferrin saturation (TSAT) < 20% and ferritin < 100 ng/mL. Children were classified into four groups: IDA, non-anemia iron deficiency (NAID), control and others. Results: Out of 970 children, 332 (34.2%) had NAID and 278 (28.7%) presented with IDA. Analysis revealed that Ret-He significantly correlates with ferritin (rho = 0.41; *p* < 0.001), TSAT (rho = 0.66; *p* < 0.001) and soluble transferrin receptor (sTfR) (rho = −0.72; *p* < 0.001). For ROC analysis, the area under the curve (AUC) was 0.771 for Ret-He detecting ID and 0.845 for detecting IDA. The cut-off value for Ret-He to diagnose ID was 33.5 pg (sensitivity 90.7%; specificity 35.8%) and 31.6 pg (sensitivity 90.6%; specificity 50.4%) to diagnose IDA. Conclusions: The present study demonstrates Ret-He to be a screening marker for ID and IDA in children. Furthermore, Ret-He can be used as a single screening parameter for ID and IDA in children without considering other iron parameters. Economically, the use of Ret-He is highly relevant, as it can save one blood tube per patient and additional costs.

## 1. Introduction

Iron deficiency (ID) is one of the most common nutritional deficiencies in children worldwide. In many cases, ID erythropoiesis results in iron deficiency anemia (IDA). In the pediatric population, the prevalence of anemia is highest (47.4%) in children of preschool age (5–15 years) [1]. The early recognition of ID is crucial in order to prevent impaired cognitive performance and systemic complications arising from states of ID and/or IDA [2,3]. 

Overall, anemia in children can be classified according to the World Health Organization (WHO), depending on hemoglobin (Hb) level, age and gender [4]. The detection of anemia, such as in a surgical setting, is especially important for anesthesiologists with respect to improve patients’ outcome. 

The preoperative identification and treatment of ID with or without anemia is a cornerstone of Patient Blood Management (PBM). In context of PBM, the diagnosis of ID and/or IDA in adults and in children is recommended by measurement of ferritin and transferrin saturation (TSAT) [5]. The treatment of IDA can easily be conducted by supplementation of oral or, if fast responses are required, by intravenous iron. Literature reveals that surgical patients with iron supplementation until 21 days prior to surgery benefit from improved patient outcome and increased Hb levels after surgery [6]. According to the most recent recommendations and guidelines from the National Institute for Health and Care Excellence, iron administration should be considered prior to surgery for patients who suffer from IDA [7]. Blood loss can reach critical values, especially in children, who might require blood transfusions at low volumes. In addition, blood transfusions result in immunological responses and should be avoided. The treatment of ID/IDA with iron is a goal directed, straight forward therapy.

Overall, IDA is detected relatively late by classic laboratory parameters such as Hb, mean corpuscular volume (MCV) and mean corpuscular hemoglobin (MCH). A single biomarker is rarely used for the diagnosis of IDA or non-anemia iron deficiency (NAID), but the use of a marker that can be easily used as a screening marker is necessary. The lifespan of circulating erythrocytes is about 120 days. Changes in MCV and Hb values occur at a later time point when IDA is already fulminant [8]. Reticulocytes are formed in bone marrow and develop into mature erythrocytes within two days in the peripheral blood [9]. Therefore, the determination of Hb content in reticulocytes (Ret-He; reticulocyte hemoglobin equivalent) provides early information about the current iron availability in erythropoiesis [10].Ferritin and TSAT are often used for the diagnosis of ID. However, due to its nature as an acute phase protein, ferritin levels may increase in the presence of inflammation, whereas the Ret-He value remains low [11]. 

In adults, the Ret-He has already been proposed as an additional marker to screen for ID [8,11,12,13,14,15,16]. For example, Toki et al. revealed that Ret-He may be associated with ID by a sensitivity of 92.0% and a specificity of 81.0% (area under the curve (AUC) = 0.902) using a Ret-He cut-off value of ≤30.9 pg in adults [17]. In another study, Ret-He ≤ 25.4 pg predicted ID in adults with a sensitivity of 90.4% and a specificity of 49.1% [18]. Screening for ID and IDA using Ret-He in context of PBM has been poorly studied, especially in the pediatric surgical population. The parameter Ret-He has already been described and used in 1999 in adults and 2006 in children [19,20,21,22,23]. We now investigate the usefulness of Ret-He for screening ID and IDA in a large pediatric population. Therefore, the present study aims to evaluate the validity of Ret-He in context of PBM to screen for ID in children. 

## 2. Materials and Methods

### 2.1. Patients

This retrospective study was approved by the Ethics Committee of the University Hospital Frankfurt, Goethe University, Germany (Ref: 19–338), and has been performed in accordance with the Declaration of Helsinki. The requirement for written informed consent by patients was waived by the Ethical Committee. This manuscript adheres to the applicable Strengthening the Reporting of Observational studies in Epidemiology (STROBE) guidelines.

Between 1 January and 31 December 2019, hematological parameters (Hb, MCV, MCH and red cell distribution width (RCDW) and serum iron parameters (serum ferritin, TSAT, transferrin, serum iron, soluble transferrin receptor (sTfR) and Ret-He) from children between >6 months and <18 years of age were retrospectively extracted from the hospital’s medical and laboratory records. Study population includes in-patient and out-patient children from any pediatric discipline. Laboratory examination was performed at any time during the in-hospital stay.

### 2.2. Classification of Anemia and Iron Deficiency

In this study, anemia was defined according to the WHO definition for children that is irrespective of gender in children aged 6–59 months with a Hb level < 11.0 g/dL, children aged 5–11 years with Hb < 11.5 g/dL and children aged 12–14 years with Hb < 12.0 g/dL. For children at the age of ≥15 years, anemia was defined as Hb < 12.0 g/dL in female children and Hb < 13.0 g/dL in male children [4].

Iron deficiency was defined by TSAT < 20% and serum ferritin level < 100 ng/mL, according to Munoz [24,25], Anker et al. [26] and the diagnosis and treatment of iron deficiency guidelines [27,28,29]. TSAT assesses the iron availability to tissues, and a low TSAT (<20%) indicates inadequate iron supply to support normal erythropoiesis. The combination of TSAT and ferritin with the given limits is considered valid [25]. The European Consensus on the Diagnosis and Treatment of Iron Deficiency and Anemia in Inflammatory Bowel Disease (ECCO Guidelines) recommends iron supplementation for patients with TSAT < 20% and additional ferritin levels < 100 ng/mL [27]. Guidelines for the pre-operative optimization of patients with anemia also specify this combination as a threshold [28,29]. Non-anemia iron deficiency was defined by TSAT < 20% and a serum ferritin level < 100 ng/mL and Hb level above the WHO definition of anemia. Iron deficiency anemia was defined by TSAT < 20%, serum ferritin level < 100 ng/mL and Hb level below the WHO definition of anemia. 

### 2.3. Laboratory Parameters

All of the patients’ data were automatically extracted from the electronic laboratory information system, swisslab (Nexus swisslab GmbH, Berlin, Germany), version 2.18.1 until November 2019; swisslab II (Nexus swisslab GmbH, Berlin, Germany), version 2.21.6 from December 2019. Hemoglobin was measured using the XN-1000 (Sysmex, Norderstedt, Germany). Ret-He was measured by fluorescence flow cytometry in the reticulocyte channel of the XN-1000 (Sysmex, Norderstedt, Germany). MCV, MCH and RCDW were calculated automatically by the XN-1000 (Sysmex, Norderstedt, Germany). MCH was calculated using the following formula: (hematocrit (%)/red blood cell (×10^6^/µL)) × 10. MCV was calculated using the following formula: (Hb (g/dL)/red blood cell (×10^6^/µL)) × 10. RCDW was calculated using the following formula: (1 SD/MCV) × 1000. The following parameters were measured by using Roche instruments (Roche Diagnostics, Mannheim, Germany); serum iron (cobas 8000 c701); ferritin (cobas 8000 e801); and sTfR (cobas 8000 c502). Transferrin saturation was calculated as follows: (serum iron (mg/dL)/transferrin (mg/dL)) × 70.9 [30].

### 2.4. Primary Endpoint

Primary endpoint was the validation of Ret-He as a screening marker for ID and IDA in children.

### 2.5. Secondary Endpoints

Secondary endpoints were the children’s Ret-He levels in the four groups and correlation between Ret-He and ferritin or TSAT.

### 2.6. Statistical Analysis

According to Hb level and iron status, four groups were formed for preliminary analyses: the IDA group (anemia plus ID), NAID group (no anemia but ID; non-anemia iron deficiency), control group (no anemia; no ID) and group of others (anemia due to any other reasons; no ID). 

Data are presented as mean ± standard deviation (SD) or median and interquartile range (IQR) (25%; 75%). Boxplots were used to illustrate data distribution. The Shapiro–Wilk test was performed to assess the normality of continuous variables. As all continuous variables were non-normally distributed, groups were compared by using the Kruskal–Wallis test. Then, a post hoc test (Dunn’s test) was applied to assess differences between the groups. Spearman’s correlation was used to investigate the correlation between Ret-He and ferritin or TSAT. A *p*-value of <0.05 was considered statistically significant.

In addition, Receiver-operating characteristic (ROC) analysis was performed to assess the potential capabilities of Ret-He as a marker for ID (with and without anemia) and IDA, computing the ROC curve and its AUC (with 95% confidence interval (CI)). The results of this analysis can be shown as the ROC curve, in which the sensitivity is plotted against the value of 1-specificity. If the AUC is almost 1, the accuracy of the test should be considered excellent. The objective is to find a cut-off value for Ret-He for ID and IDA in children as a screening parameter. Therefore, a sensitivity of 90% was defined as the threshold.

All statistical analyses and graphical illustrations were performed by using R© (Version 3.6.3, R Foundation for Statistical Computing, Vienna, Austria) and IBM^®^SPSS^®^ Statistics (Version 26, IBM, Armonk, NY, USA).

## 3. Results

### 3.1. Patients’ Characteristics

Between 1 January 2019 and 31 December 2019, data from 972 cases were identified. Of these, two cases were excluded due to incomplete medical records. In total, 970 cases were included in the final analysis. 

Overall, there were 506 (52.2%) male and 464 (47.8%) female children. The mean age was 11.2 (±4.9) years. There were 278 (28.7%) children in the IDA group, 332 (34.2%) children in the NAID group, 302 (31.1%) children in the control group and 58 (6.0%) children in the group of others. As expected, the median Hb level was the lowest in IDA children (10.5 (9.6; 11.3) g/dL) compared with NAID children (12.6 (12.1; 13.5) g/dL), children in the control group (13.3 (12.6; 14.1) g/dL) and children in the group of others (10.8 (9.4; 11.7) g/dL) (Table 1). In Table 1, other laboratory parameters such as Ret-He, ferritin, TSAT, serum iron, transferrin, sTfR, MCH, MCV and RCDW are listed for all four groups.

### 3.2. Comparison of Ret-He Values According to the Iron Status

**Figure 1:**
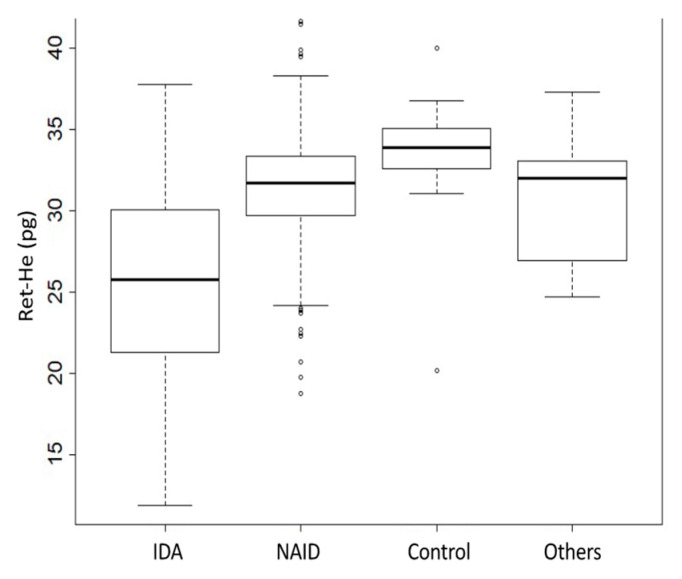
Ret-He values in different patient groups. Figure 1 represents the Ret-He values among the four patient groups. The group of others includes anemia due to any other reason and no ID. Comparison of values was performed using the Kruskal–Wallis Test. Dunn’s test was applied post hoc to assess the differences between the groups of interest; *p* < 0.001 for comparing IDA vs. NAID; *p* < 0.001 for IDA vs. Control; *p* < 0.001 for NAID vs. Control. Ret-He, reticulocyte hemoglobin equivalent; IDA, iron deficiency anemia; NAID, non-anemia iron deficiency.

The median Ret-He values were significantly lower in the IDA group (25.2 (20.5; 29.1) pg) compared to the NAID group (30.7 (28.6; 32.5) pg; *p* < 0.001) or the control group (32.8 (31.4; 34.2) pg; *p* < 0.001). In addition, the differences in Ret-He values differed significantly between the NAID group and the control group (*p* < 0.001) (Figure 1).

### 3.3. Relation of Ret-He with Parameters of Iron Metabolism

**Figure 2:**
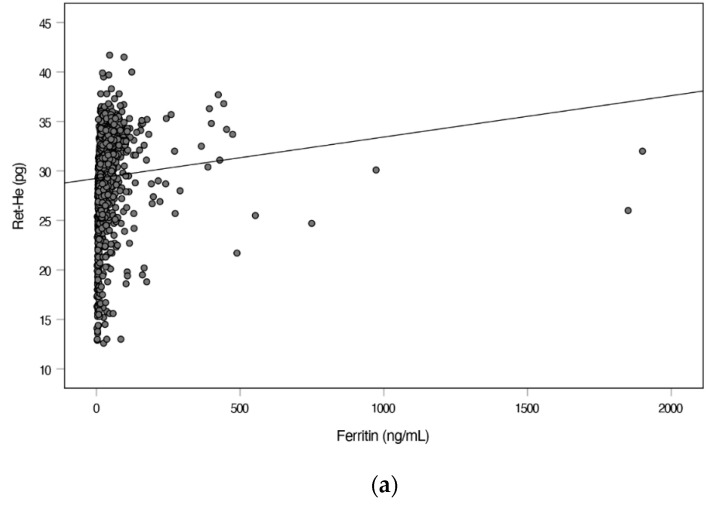

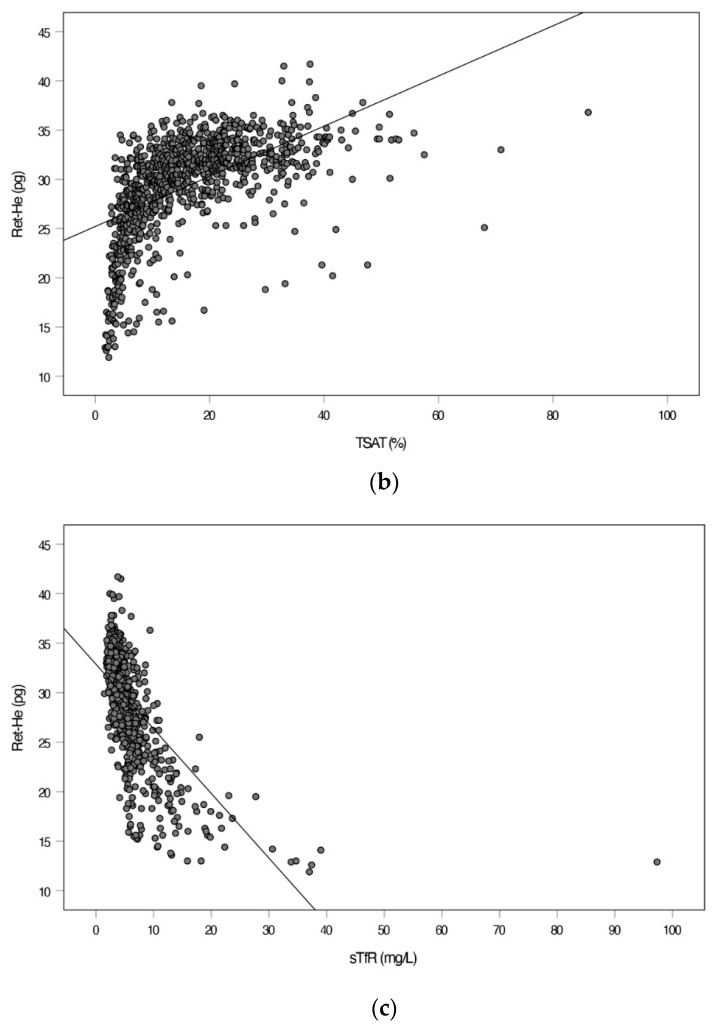
Relation between (**a**) Ret-He and ferritin, (**b**) Ret-He and TSAT and (**c**) Ret-He and sTfR in all patients. Figure (**a**) depicts the correlation between Ret-He and ferritin. *p*-value < 0.001 for the spearman correlation coefficients rho of Ret-He and Ferritin. Figure (**b**) depicts the correlation between Ret-He and TSAT. *p*-value < 0.001 for the spearman correlation coefficients rho Ret-He and TSAT. Figure (**c**) depicts the correlation between Ret-He and sTfR. *p*-value < 0.001 for the spearman correlation coefficients rho Ret-He and sTfR. TSAT, transferrin saturation; Ret-He, reticulocyte hemoglobin equivalent; sTfR, soluble transferrin receptor.

As presented in Table 1, ferritin was lowest in IDA children, followed by NAID children. Other forms of anemia (e.g., anemia of inflammation or anemia of chronic disease) presented with increased ferritin levels, as expected due to the acute phase reactions resulting in an induction of ferritin. We correlated Ret-He with ferritin and found a significant positive correlation with ferritin (Spearman’s correlation coefficient rho = 0.41; *p* < 0.001) (Figure 2a).

Another relevant parameter for the diagnosis of ID is TSAT. In the pediatric cohorts, controls presented with values within the normal range but with a wide span. By contrast, IDA patients had the lowest TSAT values, followed by NAID patients. In pediatric patients with anemia caused by other factors, including anemia of inflammation, values were higher than the normal range, as expected due to the inflammatory decrease in transferrin, which increased TSAT. The correlation of Ret-He with TSAT was high (rho = 0.66; *p* < 0.001) (Figure 2b). Further analysis demonstrated a significant strong correlation between Ret-He and sTfR (rho = −0.72; *p* < 0.001) (Figure 2c).

### 3.4. ROC Analysis

**Figure 3:**
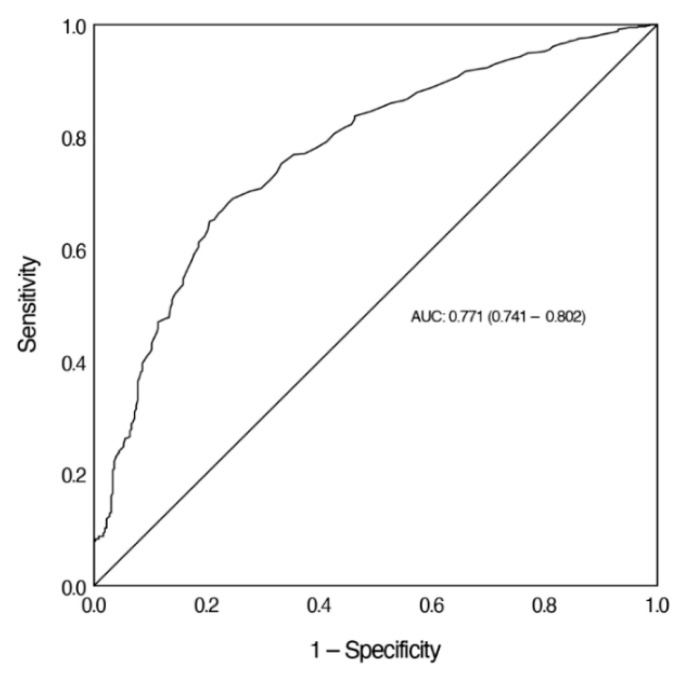
ROC analysis of Ret-He to screen for ID. Figure 3 demonstrates the ROC curve of Ret-He in the diagnosis of ID. Ret-He, reticulocyte hemoglobin equivalent; AUC, area under the curve.

The diagnostic accuracy of Ret-He in screening for ID was assessed by ROC analysis. The AUC for Ret-He to screen for ID was 0.771. We calculated the sensitivity of Ret-He at a sensitivity of more than 90%. For a sensitivity of 90.7% and a specificity of 35.8%, the cut-off value of Ret-He was 33.5 pg (Figure 3).

**Figure 4:**
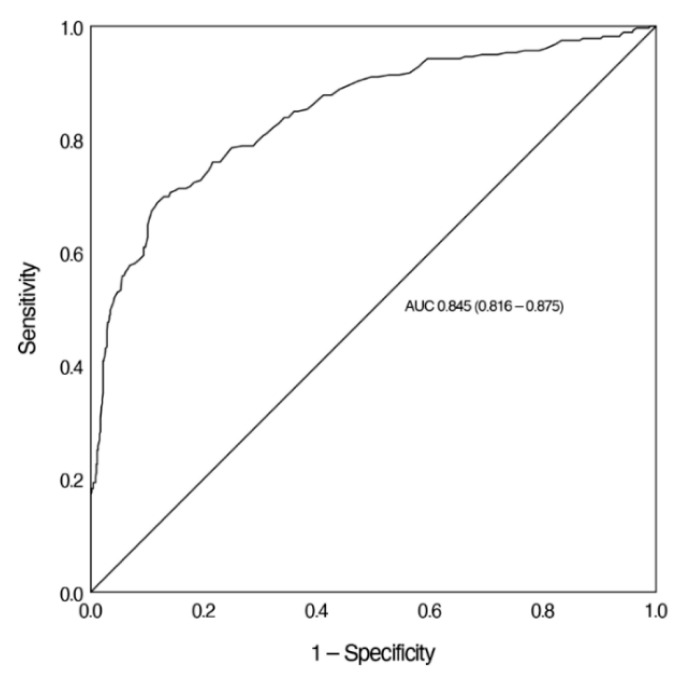
ROC analysis of Ret-He to screen for IDA. Figure 4 demonstrates the ROC curve of Ret-He in the diagnosis of IDA. Ret-He, reticulocyte hemoglobin equivalent; AUC, area under the curve.

The AUC for Ret-He to screen for IDA was 0.845. Again, we calculated the sensitivity of Ret-He at a sensitivity of more than 90%. For a sensitivity of 90.6% and a specificity of 50.4%, the cut-off value of Ret-He was 31.6 pg (Figure 4).

Therefore, the present data indicate that Ret-He may be an effective marker with sufficient sensitivity to screen for ID and IDA in children. 

## 4. Discussion

This study evaluated the validity of Ret-He as a screening marker for ID and IDA in children between >6 months and <18 years of age. The analysis revealed that children with IDA had significantly lower Ret-He levels than children with NAID or children with normal iron and Hb values. In addition, Ret-He correlated positively with common laboratory parameters for ID, including ferritin and TSAT. As a result of the ROC analysis, this study shows the validity for using Ret-He to screen for ID (AUC = 0.771) and IDA (AUC = 0.845). 

Due to the retrospective study design of the present study, some disadvantages are obvious. However, the data were queried and evaluated according to standardized methods. It has to be mentioned that pathophysiological reasons and treatments causing states of ID and IDA were not analyzed. 

However, this study represents one of the largest pediatric patient populations at a PBM center in order to evaluate the usefulness of Ret-He as a screening marker for ID and IDA in children. The context of PBM screening for iron depleted states in children is especially crucial, as iron supplementation may be considered if indicated. In addition, any associated acute or chronic medical aspect was not considered in the present study. Therefore, children with hemoglobinopathies (e.g., α-thalassemia or β-thalassemia) were not identified and included in our analysis. In order to address the usefulness of Ret-He, especially in children with red blood cell disorders such as thalassemia, this particular patient cohort should be considered in future studies. A challenge are times of the blood draws and laboratory examinations during the children’s hospital stay, as they were not available and could not be correlated. 

Overall, parameters such as ferritin and TSAT are widely used in the context of ID. Nevertheless, recent studies have demonstrated that Ret-He may also be advantageous for the screening of ID, because it can be easily obtained and is free from biological variability [31,32]. Due to the short duration of the reticulocyte stage in erythropoiesis, the evaluation of Ret-He can reveal states of ID that are clinically significant, but not yet reflected by hematological parameters such as Hb, MCV and MCH [8].

In our study, the comparison of Ret-He levels between the patient groups revealed that children in the IDA group had significantly lower Ret-He levels than those in the control group. Moreover, data indicated that the decrease in RET-He was dependent on the severity of ID, which may lead to states of IDA. These results suggest that Ret-He reflects the iron state of children and may have potential as a useful screening marker for ID. Of note, Ret-He may also be low in hemoglobinopathies such as α-thalassemia or β-thalassemia. A retrospective analytical study by Chinudomwong et al. analyzed 253 children with IDA and 276 children with thalassemia. The results revealed that there was no significant difference in Ret-He values between children with IDA and thalassemia (median 20.6 pg (IQR) 9.0–19.6 pg; *p* = 0.57). Serum ferritin levels differed between IDA and thalassemia (*p* < 0.0001). The authors suggest that upon diagnosis of iron depletion by Ret-He with potential diagnosis of thalassemia, serum ferritin should be determined. Especially in countries with high thalassemia prevalence, the usefulness of Ret-He to distinguish between IDA and hemoglobinopathy carriers has to be studied in future trials [11]. 

Furthermore, the comparison of Ret-He in all patients with standard parameters of iron metabolism (ferritin and TSAT) showed significant positive correlations of Ret-He with ferritin and TSAT. These results are in line with a previously published study of 100 children suffering from cancer. The authors also detected a correlation between Ret-He and ferritin in a subgroup analysis in patients with ID [33]. Analysis revealed a strong correlation of Ret-He with sTfR. Thus far, there has been a limited number of studies regarding the validity of Ret-He in children with ID [9,19,20,21,22]. In addition, the reference values of Ret-He as a screening marker for ID remain unclear.

In this study, ROC analysis revealed a valid utility of Ret-He in screening for ID (AUC = 0.771) and IDA (AUC = 0.845). For pediatric patient groups, cut-off values between 26.0 pg and 29.0 pg have been reported with varying sensitivity and specificity (Table 2) [20,21,23,34]. In adults, the use of Ret-He is strongly recommended in states of ID [16,28]. In this situation, a cut-off value of 30.9 pg is considered an indicator of ID, with a high corresponding sensitivity of 92.0% and a specificity of 82.0% (AUC = 0.902) [17]. Compared with the abovementioned studies, the concomitant existence of high sensitivity and specificity (both over 90%) for Ret-He as a screening marker for ID could not be achieved in our study. At this point, it has to be highlighted that the present study was conducted in order to evaluate the usefulness of Ret-He in screening for ID and IDA in children and not for diagnosis. According to the primary endpoint of Ret-He as a screening marker for ID and IDA in children, screening usually requires high sensitivity. False positive results are possible with low specificity; therefore, a subsequent differentiated anemia diagnostic is necessary. In addition, the cited studies used only one Hb limit to define anemia in the ROC analysis for all age groups, whereas in the present study an age-group-adjusted Hb value (following the WHO recommendations for anemia) was considered. When calculated based on an Hb level limit (Hb level below 11 g/dL), our analysis would yield the following results: AUC 0.925 with specificity 94.9% and sensitivity 67.4%. In a future, prospective trial, these age groups could be separately analyzed. From a statistical perspective, there seems to be no standard policy defined if either a higher sensitivity or a higher specificity is needed in screening for ID, or if both measures should fall within a certain average value range.

Iron deficiency anemia can be treated with intravenous or oral iron supplementation. A study on 65 hemodialysis patients with IDA and intravenous iron administration revealed that changes in Ret-He during iron treatment after two weeks (*p* < 0.001) and 4 weeks (*p* < 0.001) strongly correlated with response to intravenous iron supplementation compared to baseline values [35]. Furthermore, it has been demonstrated that in patients with IDA and oral iron administration, the increase in Ret-He mirrors the increase in Hb in response to treatment [17]. Therefore, Ret-He may be an effective parameter for evaluating responses to iron treatment in adults and children with IDA.

The costs of Ret-He measurement are of great clinical and economical relevance; Ret-He is inexpensive and can be easily measured in the same blood tube of cellular blood analysis, with costs of approximately EUR 0.70–1.00 per measurement [36]. This is lower than standard costs for traditional ferritin and TSAT parameters [31]. It is noteworthy, that there are different instruments for the measurement of Ret-He. In our study, Ret-He was measured by fluorescence flow cytometry using the XN-1000 series by Sysmex (Norderstedt, Germany). Ret-He can also be measured by the automated flow cytometer of Technicon H*3 (Bayer Diagnostics, Tarrytown, NY, USA) [23] or the ADVIA-120 counter (Siemens Healthcare Diagnostics, NY, USA) [20].

In our study, we were able to show that the screening for IDA is possible solely based on Ret-He. For the educational aspects regarding the screening of IDA in the context of PBM, the use of Ret-He may be strengthened. As ID is the most common nutritional deficiency in children, effort should focus on early treatment to prevent neurodevelopmental and behavioral impairments [2]. Screening for ID and IDA may not be easy as it is based on several hematological and iron metabolism parameters. However, analysis reveals that Ret-He is a useful screening marker, not for diagnosis, but for iron-depleted states in children without the necessity of further parameters and a parameter, that can be determined from the same blood tube saving twice, blood and costs. Lastly, Ret-He could be helpful when screening for IDA in children suffering from inflammatory disorders in which IDA co-exists.

## 5. Conclusions

The present study demonstrates Ret-He to be a valid screening marker for ID and IDA in children but not for diagnosis. Furthermore, Ret-He can be used as a single screening parameter for ID and IDA in children without considering other iron parameters. However, future prospective studies have to be conducted in order to demonstrate the clinical benefit and additional cost benefit of Ret-He. In addition, analyses of Ret-He in combination with other hematological parameters (e.g., MCH or MCV) may be of high interest in future studies. Economically, the use of Ret-He in screening for ID and IDA is highly relevant as it can save one blood tube per patient and additional costs.

## Figures and Tables

**Table 1:** Demographic data and laboratory results of study population.

	All Patients *n* (%) 970 (100.0)	IDA *n* (%) 278 (28.7)	NAID *n* (%) 332 (34.2)	Control *n* (%) 302 (31.1)	Others *n* (%) 58 (6.0)
Age (years) * ^#^	11.2 (±4.9) 12.0 (7.0; 15.0)	11.3 (±5.1) 13.0 (7.3; 16.0)	10.3 (±4.9) 11.0 (6.0; 15.0)	11.9 (±4.3) 13.0 (8.3; 16.0)	11.8 (±5.3) 14.0 (6.5; 16.0)
Male *n* (%)	506 (52.2)	147 (52.9)	180 (54.2)	151 (50.0)	28 (48.3)
Hb (g/dL) ^#^	12.3 (11.2; 13.3)	10.5 (9.6; 11.3)	12.6 (12.1; 13.5)	13.3 (12.6; 14.1)	10.8 (9.4; 11.7)
Ret-He (pg) ^#^	30.8 (27.2; 32.9)	25.2 (20.5; 29.1)	30.7 (28.6; 32.5)	32.8 (31.4; 34.2)	30.2 (25.8; 33.5)
Ferritin (ng/mL) ^#^	27 (13; 49)	11.0 (7.0; 23.82)	27 (16; 42)	42.5 (27.0; 65.8)	75.0 (23.0; 198.0)
TSAT (%) ^#^	14.4 (77.7; 22.5)	6.0 (4.1; 9.9)	12.8 (9.0; 16.2)	26.1 (22.3; 33.0)	21.8 (20.9; 28.3)
Serum Iron (µg/dL) ^#^	58 (31; 91)	27.0 (18.0; 40.0)	53.0 (38.0; 67.0)	103.0 (88.0; 123.8)	89.5 (45.0; 111.8)
Transferrin (mg/dL) ^#^	292 (259; 324.8)	315.5 (288; 355.8)	295 (268.8; 324.0)	272.0 (250.0; 305.0)	240.5 (191.8; 279.0)
sTfR (mg/L) ^#^	4.0 (3.1; 5.6)	6.5 (4.7; 10.2)	4.2 (3.4; 5.0)	3.1 (2.6; 3.6)	4.0 (3.0; 5.8)
MCH (pg) ^#^	26.8 (24.3; 28.4)	22.9 (20.4; 25.4)	27.0 (25.4; 28.3)	28.4 (27.2; 29.5)	25.3 (23.1; 27.6)
MCV (fl) ^#^	78.2 (73.1; 82.2)	72.1 (66.2; 77.4)	78.3 (74.8; 81.9)	81.6 (78.4; 84.8)	76.2 (71.9; 80.4)
RCDW (%) ^#^	13.5 (12.5; 15.4)	16.1 (14.6; 18.8)	13.2 (12.5; 14.1)	12.5 (12.1; 13.2)	15.5 (13.6; 18.4)

Table 1 shows the demographic data and laboratory results of the study population. Results are expressed as mean (±SD) * and median (IQR) ^#^. IDA, iron deficiency anemia; NAID, non-anemia iron deficiency; Hb, hemoglobin; MCV, mean corpuscular volume; MCH, mean corpuscular hemoglobin; TSAT, transferrin saturation; sTfR, soluble transferrin receptor; Ret-He, reticulocyte hemoglobin equivalent; RCDW, red cell distribution width; g, gram; dL, deciliter; µg, microgram; mg, milligram; ng, nanogram; pg, picogram; fl, femtoliter.

**Table 2:** Study results for cut-off values for the diagnosis of iron deficiency in children.

Patients	*n*	Cut-Off-Value	AUC	Sensitivity	Specificity	Reference
Children on chronic dialysis	45	28.9 pg	0.870	90%	75%	Davidkova et al. [21]
Healthy 9–12 months old infants	202	27.5 pg	0.850	83%	72%	Ullrich C et al. [20]
General pediatric outpatient	210	26.0 pg	*	70%	78%	Brugnara et al. [23]
Children on hemodialysis	40	29.0 pg	*	90%	75%	Di Pinto et al. [34]

Table 2 shows the cut-off values for Ret-He reported for screening of ID in children. * No AUC data included in the study. AUC; Area under the curve.

## Data Availability

The data that support the findings of this study are available from the corresponding author upon reasonable request.
